# The structural and functional effects of the familial hypertrophic cardiomyopathy-linked cardiac troponin C mutation, L29Q

**DOI:** 10.1016/j.yjmcc.2015.08.017

**Published:** 2015-10

**Authors:** Ian M. Robertson, Ivanka Sevrieva, Monica X. Li, Malcolm Irving, Yin-Biao Sun, Brian D. Sykes

**Affiliations:** a Randall Division of Cell & Molecular Biophysics, King's College London, London SE1 1UL, UK; b British Heart Foundation Centre of Research Excellence, King's College London, London SE1 1UL, UK; c Department of Biochemistry, Medical Sciences Building, University of Alberta, Edmonton, Alberta T6G 2H7, Canada

**Keywords:** TnC, troponin C, cTnC, cardiac TnC, cNTnC, N-terminal domain of cTnC, cTnI, cardiac troponin I, cTnT, cardiac troponin T, sNTnC, N-terminal domain of skeletal TnC, ScTnC, trout cTnC, NMR, nuclear magnetic resonance, CD, circular dichroism, NOE, nuclear overhauser enhancement, NOESY, NOE spectroscopy, RMSD, root-mean-square deviation, FISS, fluorescence for in situ structure, BR, bifunctional rhodamine, PKA, protein kinase A, PKC, protein kinase C, HCM, hypertrophic cardiomyopathy, FHC, familial hypertrophic cardiomyopathy, Troponin C, Hypertrophic cardiomyopathy, L29Q, NMR spectroscopy, Fluorescence spectroscopy, Cardiac

## Abstract

Familial hypertrophic cardiomyopathy (FHC) is characterized by severe abnormal cardiac muscle growth. The traditional view of disease progression in FHC is that an increase in the Ca^2 +^-sensitivity of cardiac muscle contraction ultimately leads to pathogenic myocardial remodeling, though recent studies suggest this may be an oversimplification. For example, FHC may be developed through altered signaling that prevents downstream regulation of contraction. The mutation L29Q, found in the Ca^2 +^-binding regulatory protein in heart muscle, cardiac troponin C (cTnC), has been linked to cardiac hypertrophy. However, reports on the functional effects of this mutation are conflicting, and our goal was to combine *in vitro* and *in situ* structural and functional data to elucidate its mechanism of action. We used nuclear magnetic resonance and circular dichroism to solve the structure and characterize the backbone dynamics and stability of the regulatory domain of cTnC with the L29Q mutation. The overall structure and dynamics of cTnC were unperturbed, although a slight rearrangement of site 1, an increase in backbone flexibility, and a small decrease in protein stability were observed. The structure and function of cTnC was also assessed in demembranated ventricular trabeculae using fluorescence for *in situ* structure. L29Q reduced the cooperativity of the Ca^2 +^-dependent structural change in cTnC in trabeculae under basal conditions and abolished the effect of force-generating myosin cross-bridges on this structural change. These effects could contribute to the pathogenesis of this mutation.

## Highlights

• The cTnC L29Q mutation causes a small change in the NMR structure of site 1 in cTnC.
• L29Q reduces the cooperativity of Ca^2 +^-dependent structural changes in cTnC *in situ*.
• L29Q removes the impact of force-generating myosin heads on cTnC structural changes.

## Graphical abstract

**Figure:**
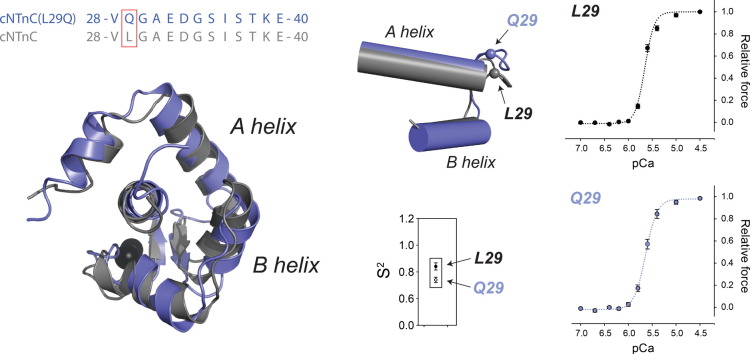

## 1 Introduction

Heart muscle contraction is regulated by the Ca^2 +^-binding protein complex troponin. Troponin is a heterotrimeric protein complex that, with its regulatory partner tropomyosin, is bound to the actin-containing thin filaments of the muscle sarcomere at regular intervals of 7-actin monomers. Cardiac troponin is comprised of: troponin C (cTnC), the Ca^2 +^-binding subunit; troponin I (cTnI), the inhibitory subunit; and troponin T (cTnT), the tropomyosin binding subunit. During muscle contraction, Ca^2 +^ is released to the cytosol and binds to the low affinity Ca^2 +^-binding site (site 2) of the N-terminal domain of cTnC (cNTnC). With Ca^2 +^-association, cNTnC binds to the switch region of cTnI (cTnI_147–163_), which in-turn drags the inhibitory and mobile domains of cTnI off the actin thin filament. Once cTnI dissociates from actin, the position of tropomyosin changes, exposing the myosin head binding sites. Following myosin head attachment, the power stroke pulls the thin filament towards the M-line at the centre of the sarcomere, causing the cardiomyocyte to shorten and muscle contraction to occur. Muscle relaxation occurs when cytosolic Ca^2 +^ levels decrease, resulting in a dissociation of Ca^2 +^ and the switch region of cTnI from cNTnC. The inhibitory and mobile domains of cTnI bind to actin, triggering a rearrangement of tropomyosin along the thin filament and a resultant blocking of the myosin-head binding sites. For reviews on the mechanism of regulation of contraction see Refs [1], [2], [3].

Familial hypertrophic cardiomyopathy (FHC) is an inherited disorder that is characterized by left ventricle hypertrophy that develops into heart failure and arrhythmia [4], [5]. Approximately 1 in 500 adults have FHC, which makes it the most common genetic cardiovascular disease [6], [7]. While FHC mutations have been identified across a wide range of proteins, more than half of all mutations that cause hypertrophy occur in proteins of the sarcomere [5]. As of 2010, 68 mutations in the troponin complex had been linked to FHC [4]. The first mutation associated with FHC in cTnC was L29Q (cTnC(L29Q)), which was identified in a patient suffering from left ventricular hypertrophy [8].

In the context of the structure and function of cTnC, the location and hydrophobic-to-hydrophilic nature of L29Q is provocative. L29 lies at the start of the defunct Ca^2 +^-binding site in cNTnC (site 1). In the regulatory N-terminal domain of skeletal troponin C (sNTnC), both EF-hands bind Ca^2 +^ (at sites 1 and 2). Ca^2 +^-binding to sNTnC induces an opening of the domain that exposes a central hydrophobic patch [9], which forms its interface with the switch region of troponin I [10]. On the other hand, as a result of an insertion at residue 28 (V28) and two amino acid replacements (D29L and D31A), site 1 of cNTnC has lost its ability to bind Ca^2 +^. Therefore, only one Ca^2 +^ binds to cNTnC (at site 2), and this produces a smaller opening than that observed in sNTnC [11], [12], [13]. In cNTnC, Ca^2 +^ binding is considered to shift the equilibrium towards the open state [14], but the fully open state is stabilized only once cTnI_147–163_ is bound [15]. Shifting the closed-to-open equilibrium further towards the open state would be a means of increasing Ca^2 +^-sensitivity of muscle contraction by increasing the affinity of cTnI_147-163_ for cNTnC. This mechanism has been exploited by the engineered L48Q mutation of cNTnC [16], [17] and by Ca^2 +^-sensitizing drugs [18], and since L29 makes hydrophobic interactions with nearby residues that stabilize the conformation of the loop in site 1 [12], [19], replacement of L29 by glutamine might be expected to produce a structural perturbation that could alter the function of cTnC.

L29 is also important in the binding of the cardiac specific N-terminus of cTnI (cTnI_1–32_) to cTnC. NMR and cross-linking studies have indicated that cTnI_1–32_ comes in close contact with cNTnC, interacting specifically with residues in the defunct site 1 (including L29) [20], [21], [22], [23]. cTnC(L29Q) is also insensitive to the Ca^2 +^ desensitization caused by phosphorylation at residues S22 and S23 in cTnI_1–32_ [24], [25]. Since phosphorylation at these two residues is known to decrease fiber contractility [26], this insensitivity to phosphorylation may be the pathogenic mechanism of the L29Q mutation.

Biochemical and physiological studies that attempted to characterize the function of cTnC(L29Q) have led to conflicting results [24], [25], [27], [28], [29], [30], [31]. Ca^2 +^ sensitivity of myofilaments reconstituted with cTnC(L29Q) has been reported to be decreased [24], [30], increased [28], [31], or unchanged [27], [29]. These discrepancies have been suggested to be at least partly due to the different model systems used in these studies (i.e. heterogeneous proteins and/or tissues) [30]; however, the same range of divergent results have also been reported for isolated protein components [25], [27], [31], [32]. While the biochemical techniques used in these reports are as varied as the myofilament preparations, the wide range of published observations suggests the effect of this mutation may be subtle or negligible. Indeed, the patient identified with the L29Q mutation showed no signs of diastolic or systolic dysfunction [8].

Structural biology has played an indispensible role in facilitating our understanding of the mechanism by which troponin regulates muscle contraction [2]. However surprisingly few structures of cTnC with disease-linked cardiomyopathies have been reported [33], [34]. The X-ray crystal structure of cNTnC(L29Q) bound to eight cadmium ions was recently solved [34]; however, since cadmium was bound to both the functional and defunct Ca^2 +^-binding sites of cNTnC(L29Q), it was difficult to make conclusions on whether L29Q caused any structural perturbations when compared to the wild-type isoform.

In this study, we combined *in vitro* solution structural biology by nuclear magnetic resonance (NMR) spectroscopy and circular dichroism (CD) with *in situ* structural biology by fluorescence for *in situ* structure (FISS) to better understand the structural and functional changes associated with the L29Q mutation in physiological conditions.

## 2 Materials and methods

### 2.1 Protein expression and purification

The DNA encoding cNTnC (residues 1–89, with mutations C35S and C84S) was inserted into the pET-3a expression vector as previously described [35]. The cysteine-free variant of cNTnC was chosen for this study to be consistent with the *in situ* fluorescence experiments (see below). The L29Q mutation was engineered using cNTnC(C35S C84S) DNA as a template with a site-directed mutagenesis kit (QuikChange purchased from Stratagene). The expression and purification of ^15^N-labeled and ^13^C,^15^N-labeled cNTnC(L29Q) and ^15^N-labeled cNTnC(C35S,C84S) were as previously described [32], [36].

Two double cysteine mutants of human cTnC(C35S, C84S)^3^ (E55C/D62C (cTnC_C_) and E95C/R102C (cTnC_E_)) with or without L29Q mutation were produced by site-directed mutagenesis, expressed in *Escherichia coli* and purified as described previously [37]. Each pair of introduced cysteines was cross-linked with a bifunctional rhodamine probe (BR) [38]: BR-cTnC_C_, BR-cTnC_E_, BR-cTnC(L29Q)_C_ and BR-cTnC(L29Q)_E_. C35 and C84 were mutated to serine residues to prevent non-specific cross-linking with the native cysteines.

### 2.2 Circular dichroism

Circular dichroism (CD) experiments were conducted on a JASCO model J-820 CD spectropolarimeter at 25 °C. The data were collected from 260 to 200 nm for the initial spectral measurements and from 230 to 210 nm for stability measurements. All measurements were made in 1 cm cuvettes with a sensitivity of 50 mDeg, a resolution of 0.5 nm, and a scan speed of 50 nm/min. The cNTnC or cNTnC(L29Q) concentrations were 2.5 μM. The buffer composition was 10 mM KH_2_PO_4_ (pH 7.1), 100 mM KCl, 1 mM EGTA, and 1 mM CaCl_2_. For the urea-induced unfolding experiments, cNTnC or cNTnC(L29Q) was dissolved in the above buffer with varying concentrations of urea (0–8 M). Urea was prepared fresh the day of the experiment. The unfolding data were fit to the following equation as previously described [39]:

$$\frac{I}{I_{0}}=y+\frac{a}{1+e^{m\left(\left[\mathrm{urea}\right]-{\left[\mathrm{urea}\right]}_{\frac{1}{2}}\right)/\mathrm{RT}}}$$

where *I* is the molar ellipticity at 222 nm ([θ]_222_) at a given urea concentration ([*urea*]), *I*_*0*_ is the initial [θ]_222_ (at 0 M urea), [*urea*]_1/2_ is the concentration of urea required to decrease the CD signal at 222 nm by half, *m* is the sensitivity towards urea unfolding, *y* and *a* are scaling constants, *R* is the ideal gas constant, and *T* is the temperature in Kelvin. The Gibbs free energy of unfolding (ΔG^0^_F-U_) in the absence of urea was calculated by multiplying *m* with [*urea*]_1/2_ [39].

### 2.3 NMR spectroscopy

All NMR experiments were run on either a Varian Inova 500-MHz or 600-MHz spectrometer equipped with a z-axis pulsed field gradient triple-resonance probes. The pulse sequences for the NMR experiments run in this study were in BioPack, the add-on package for vnmrj (Agilent Technologies). All experiments were collected at 30 °C. All NMR samples had starting volumes of 500 μL. The protein samples were dissolved in 100 mM KCl, 10 mM Imidazole, and 0.2–0.25 mM 2,2-dimethyl-2-silapentane-5-sulfonate sodium salt (DSS) (Chenomx) with 0.01% NaN_3_ in 90% H_2_O/10% D_2_O with 8 mM CaCl_2_ (Fluka). Concentrations of cNTnC(L29Q) and cNTnC were approximately 0.3–0.4 mM for all NMR experiments. The pH was monitored by the chemical shift of imidazole [40] and was kept constant between 6.6 and 6.7.

### 2.4 NMR Structure calculation

All NMR data were processed with NMRPipe [41] and visualized with NMRViewJ [42]. Backbone assignment of ^13^C,^15^N-labeled cNTnC(L29Q) was aided by the semi-automated assignment tool, Smartnotebook [43]. The NMR experiments and their experimental parameters collected for the structure determination of cNTnC(L29Q) are shown in Supplementary Table 1. Backbone dihedral angles were predicted by TALOS [44] and the HNHA experiment [45] (Supplementary Fig. 1). χ_1_ dihedral angles were determined using the HNHB [46] and HN(CO)HB [47] experiments. χ_1_ angles were assigned as being either − 60 ± 60°, 180 ± 60°, or 60 ± 60°. Interhelical angles for cNTnC(L29Q) were predicted using ORBplus [48].

Following chemical shift assignments, the automated NMR structure protocol in CYANA [49] was used to aid in the assignment of three-dimensional NOESY experiments (see Supplementary Table 1). Distance restraints were calculated in CYANA using an upper limit of 6 Å. Six distance restraints from X-ray crystallographic data for the Ca^2 +^-chelating oxygen atoms in site 2 to Ca^2 +^ and two distance restraints between D65 and G70 were included to hold the Ca^2 +^-binding loop together. After the initial calculations by CYANA, the assignments were adjusted manually and in subsequent structure calculations the manual assignments were kept during the first four CYANA calculation cycles, after which they were open for automatic assignment with the “noeassign” command of CYANA. 100 structures were calculated, and the 20 conformers with the lowest target function were used to further refine the structure. Following the CYANA refinement, peaklists were read back into NMRViewJ, confirmed manually, and converted into XPLOR-NIH [50], [51] format. The NOEs were calibrated using the median method (median distance = 3 Å) and exported as an NOE table. The simulated annealing protocol in XPLOR-NIH was used, with 10,000 high temperature steps and 6000 cooling steps. The inter-proton distances (derived from NOEs) were restrained using a soft square-well potential using the sum-averaging method. Initially, 100 structures were calculated with NOE and dihedral restraints. The 50 lowest energy structures were then refined in water with a water box edge length of 18.8 Å [52]. The final deposited ensemble (2N79.pdb) was the 20 lowest energy structures following water refinement (See Table 2 for the structural statistics).

### 2.5 ^15^N backbone relaxation experiments

The T_1_, T_2_, and NOE values were recorded for cNTnC(L29Q) and cNTnC at concentrations of 0.3–0.4 mM on a 500 MHz spectrometer. The concentrations between samples were kept as similar as possible since it has been shown that cNTnC will aggregate in a concentration-dependent manner [53]. All experiments were recorded with the same experimental parameters: T_1_ values were determined using relaxation delays of 10, 50, 100, 200, 300, 400, and 800 ms; T_2_ values were acquired using relaxation delays of 10, 30, 50, 70, 90, and 110 ms. The experiments were acquired in random order to account for field drift. The delay between transients for T_1_ and T_2_ experiments was set to 3 s. The ^1^H–^15^N NOE experiments had a delay of 3 s without the proton saturation and when proton saturation was on, it was set to 3 s. Fitting of the data was done in NMRViewJ [42]. The error was taken as the standard deviation of the background of the spectra and the peak intensities were measured using the jitter protocol. The expected correlation times (τ_m_) were calculated based on the relationship:

$$\tau_{m}=\frac{\mathrm{MW}}{2}$$

where *MW* is the molecular weight of the protein expressed in kDa and *τ*_*m*_ is in ns [54]. Model-free analysis of the data was done using Mathematica notebooks [55]. Models were chosen using Akaike's Information Criteria (AIC) [56], and Monte Carlo analysis was done on the chosen model to assess errors [57]. The model-free calculation of the order parameter (S^2^) was calculated with the S^2^-τ_m_, S^2^-τ_m_-τ_e_, S^2^-τ_m_-R_ex_, S^2^-τ_m_-τ_e_- R_ex_, and the two time-scale model (S_s_^2^-S_f_^2^-τ_m_-τ_s_). We only acquired data from 500 MHz, however, so if a good fit was obtained with the relaxation parameters in one of the simpler models it was chosen over the more complex models.

### 2.6 Reconstitution of TnC into ventricular trabeculae

Ventricular trabeculae from rat right ventricle were prepared as previously described [37]. Native TnC was partially replaced by incubation of trabeculae in relaxing solution containing 30 μmol/L BR-TnC overnight at 4 °C. The fraction of TnC replaced by BR-TnC was about 80% [58]. Following incubation, the demembranated trabeculae were mounted via aluminum T-clips between a force transducer and a fixed hook in a 60 μL trough containing relaxing solution. The sarcomere length was set to 2.1 μm. The experimental temperature was 20–22 °C.

Experimental solutions contained 25 mM imidazole, 5 mM MgATP, 1 mM free Mg^2 +^, 10 mM EGTA (except pre-activating solution), 0–10 mM total calcium, 1 mM dithiothreitol and 0.1% (*v*/*v*) protease inhibitor cocktail (P8340, Sigma). Ionic strength was adjusted to 200 mM with potassium propionate; pH was 7.1 at 20 °C. The concentration of free Ca^2 +^ was calculated using the program WinMAXC V2.5 (http://web.stanford.edu/~cpatton/maxc.html). The calculated free [Ca^2 +^] was in the range 1 nM (pCa 9) to 32 μM (pCa 4.5). In pre-activating solution, [EGTA] was 0.2 mM and no calcium was added. When required, 25 μM blebbistatin (B0560, Sigma) was added from a 10 mM stock solution in DMSO.

### 2.7 Measurement of TnC helix orientation by polarized fluorescence

Fluorescence polarization measurements were performed as previously described [37]. Briefly, a central 0.5-mm segment of a trabecula was briefly illuminated from below with 532 nm light polarized either parallel or perpendicular to the trabecular axis. BR fluorescence at 610 nm was collected both in line with the illuminating beam and at 90° to both the illuminating beam and the trabecular axis. The intensities of the parallel and perpendicular polarization components of each collected beam were used to calculate the order parameters. The order parameters <* P*_2_ > and <* P*_4_ > describe the orientation of the BR dipole, and therefore that of the helix to which it is attached, with respect to the trabecular or thin filament axis [59].

Each trabecular activation was preceded by a 1 min incubation in pre-activating solution. Isometric force and fluorescence intensities were measured after steady-state force had been established in each activation. Maximum force was recorded before and after each series of activations at submaximal [Ca^2 +^]. If the maximum force decreased by > 15%, the trabecula was discarded. The dependence of force and <* P*_2_ > on [Ca^2 +^] was fitted to data from individual trabeculae using non-linear least-squares regression to the Hill equation:

$$Y=\frac{1}{1+10^{n_{H}\left(\mathrm{pCa}-\mathrm{pC}a_{50}\right)}}$$

where *pCa*_*50*_ is the *pCa* corresponding to half-maximal change in *Y*, and *n*_*H*_ is the Hill coefficient. All values are given as mean ± standard error of the mean except where noted, with n representing the number of trabeculae.

## 3 Results

### 3.1 Structure of cNTnC(L29Q)

**Fig. 1:**
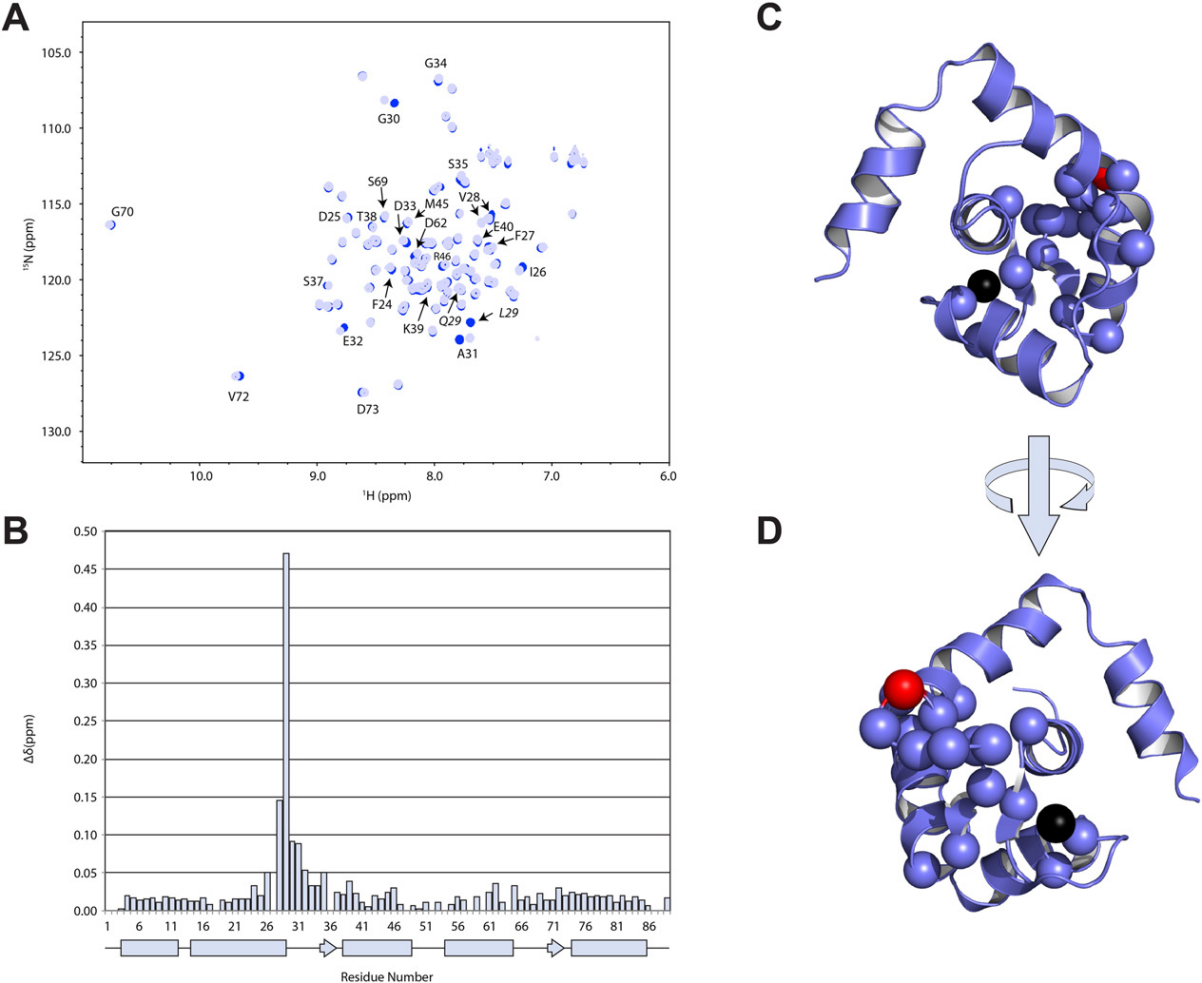
Chemical shift perturbations caused by L29Q in cNTnC. (A) An overlay of the ^1^H,^15^N HSQC spectra of cNTnC (dark blue) and cNTnC(L29Q) (light blue). (B) The chemical shift differences (Δδ) between cNTnC and cNTnC(L29Q) as a function of sequence (and secondary structure). Δδ was calculated using the formula: Δδ = ((Δδ_H_)^2^ + (0.2* Δδ_H_)^2^)^1/2^. (C and D) Residues that underwent Δδ larger than the mean are shown as spheres (Q29 is shown in red) on the cartoon representation of cNTnC(L29Q). (D) A 180° rotation about the y-axis of figure (C).

Initially, NMR chemical shift data were used to predict the global structure to assess whether L29Q altered the closed-to-open equilibrium of cNTnC. We compared the chemical shifts of cNTnC(L29Q) with the chemical shifts from a panel of cNTnC structures using the program ORBplus [48] (Supplementary Fig. 2). Principal component analysis has identified the amide chemical shifts of residues that lie in the two EF-hand loops (that include the Ca^2 +^-binding sites 1 and 2) as being the most predictive of cNTnC conformation [48]. The conformation of cNTnC(L29Q) was predicted based on residues 27–40 for the AB interhelical angle (angle between A and B helices) and residues 64–74 for the CD interhelical angle (angle between C and D helices). The closer the angle is to 180°, the more closed the structure is, whereas values closer to 90° represent a more open structure. The AB interhelical angle was predicted to be 143° and the CD interhelical angle was predicted to be 118° (Table 1). These interhelical angles are very close to those of cNTnC, indicating that the global structure of cNTnC(L29Q) is unperturbed by the mutation. Indeed, when the difference between amide chemical shifts from cNTnC and cNTnC(L29Q) are mapped on the structure of cNTnC (Fig. 1), it is evident that most of the perturbed chemical shifts are in the immediate vicinity of the mutation, indicating that a major structural change is unlikely. Therefore, we turned to high-resolution NMR spectroscopy to look for small structural perturbations caused by L29Q.

**Fig. 2:**
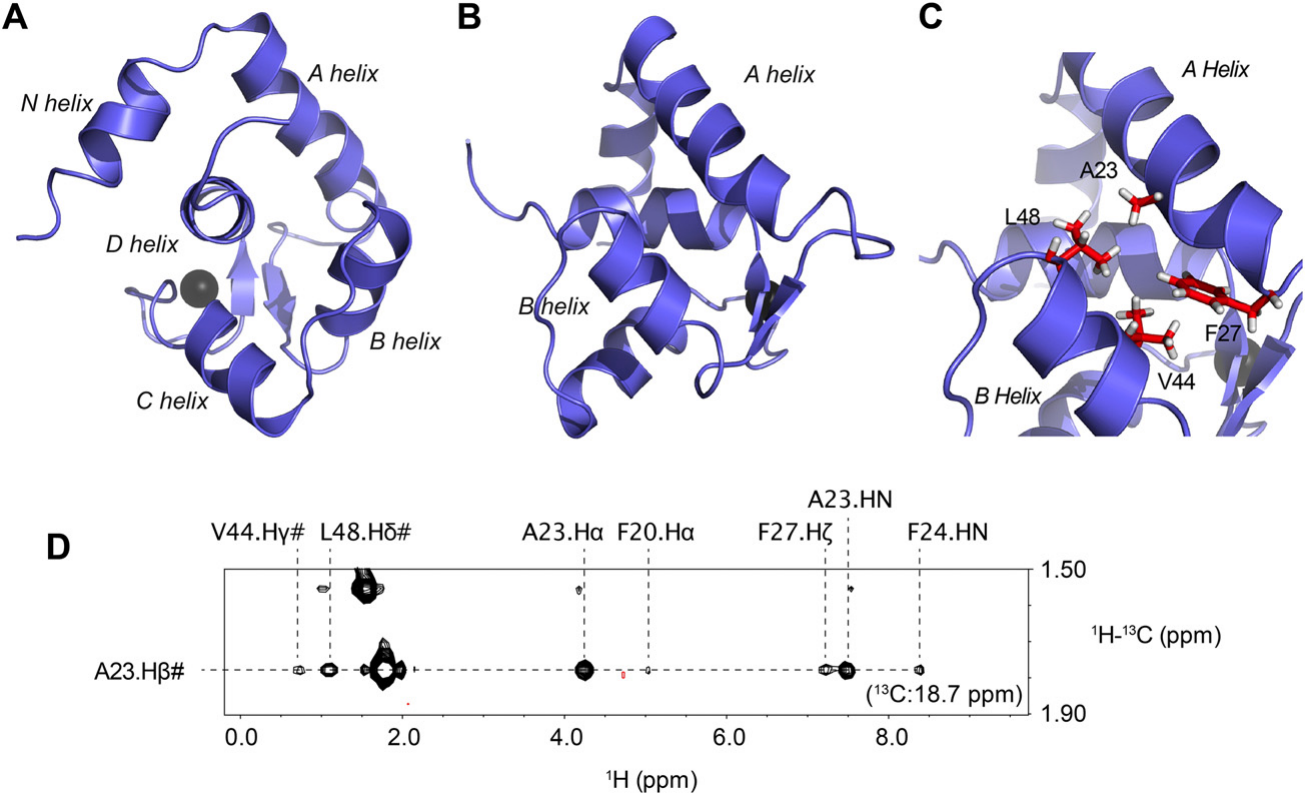
Structure of cNTnC(L29Q). A. The structure of cNTnC(L29Q) is shown in cartoon representation (Ca^2 +^ ion bound in site II as a black sphere) with the D helix pointed out of the page. B. The structure rotated by 90°. C. A close-up of the AB interhelical interface. Several of the key residues that make of this interface are shown in stick format (residues A23, F27, V44, and L48). D. NOE evidence for the closed conformation of cNTnC(L29Q). A slice from the ^13^C-HSQCNOESY spectrum highlighting the NOEs made by the methyl of A23.

The NMR structure of cNTnC(L29Q) was calculated using NMR spectroscopy (Fig. 2). 1692 distance restraints and 175 dihedral restraints (ϕ/ψ/χ_1_) were used in the refinement of the structure (structural statistics in Supplementary Table 2). The backbone Root-Mean-Square Deviation (RMSD) for residues 3–19 and 52–85 was 0.94 ± 0.18 Å. There were no ϕ/ψ/χ_1_ violations greater than 5°, no distance restraint violations > 0.3 Å, and all the ϕ/ψ angles were in the allowed regions of the Ramachandran plot. As with cNTnC, cNTnC(L29Q) contained 5 α-helices (N, A, B, C, D). There were also two small β-strands, one between helices A and B (in site 1) and one between helices C and D (in site 2), which come together to form an antiparallel β-sheet.

The three-dimensional tertiary structure of cNTnC(L29Q) was solved using distance restraints from the nuclear overhauser enhancement (NOE) spectroscopy (NOESY) NMR experiment. The NOESY experiment measures proton-proton distances within 5–6 Å, with closer proton pairs leading to stronger NOEs than nuclei that are farther apart [60]. The interhelical angles of cNTnC(L29Q), and thus its tertiary structure, depend on a few crucial interhelical NOEs. Fig. 2C and D shows a network of NOEs that were essential in determining the structure of cNTnC(L29Q). The β-methyl protons of A23 (on the A-helix) were found to make NOEs with the ζ proton of F27 (also on the A helix), as well as with the methyl protons of V44 and L48. These NOEs highlight the closed structure of cNTnC(L29Q).

### 3.2 Comparison of the structure of cNTnC(L29Q) with cNTnC and ScNTnC

**Fig. 3:**
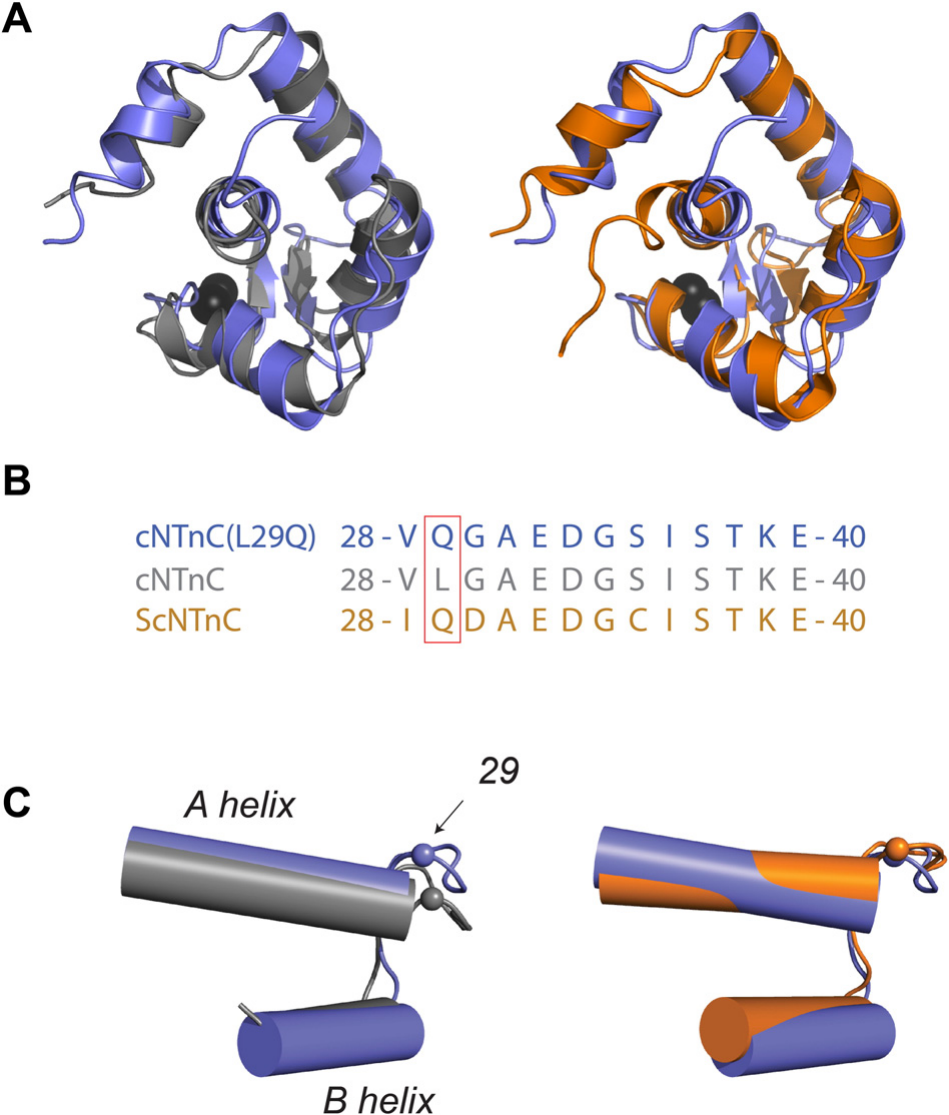
Comparison of the structure of cNTnC(L29Q) with cNTnC and ScNTnC. A. The structure of cNTnC (PDB: 2CTN, gray) and ScNTnC (PDB: 1R2U, orange) were aligned by their secondary structural elements (residues 5–10,15–27, 35–37, 40–48, 54–64, 71–73, and 74–86) to cNTnC(L29Q) (slate). B. Sequence of site 1 for cNTnC (gray), ScNTnC (orange), and cNTnC(L29Q) (slate). C. The overlay of the average structures of cNTnC(L29Q) with cNTnC (left) and ScNTnC (right) (helices are represented as cylinders). The C_α_ of residue 29 is shown as a sphere (radius set to 0.5 Å). The structures were aligned to the backbone of residues 15–27 and 41–48 and the RMSD of the C_α_ in site 1 (residues 28–40) was determined to be 3.20 Å (cNTnC) and 1.77 Å (ScNTnC). All structures are shown in cartoon representation and Ca^2 +^ ions are depicted as black spheres.

To identify whether a structural change was associated with the L29Q mutation, the structure of cNTnC(L29Q) was superimposed on that of cNTnC (Fig. 3A). The structures were aligned by their secondary structural elements (Cα atoms of residues: 5–10 (N Helix), 15–27 (A Helix), 40–48 (B Helix), 54–64 (C Helix), 74–86 (D Helix), and 35–37 and 71–73 (β-sheet)). The root-mean-square deviation (RMSD) was 1.29 Å, indicating little difference between the structures. The structure of cNTnC was characterized using the AB and CD interhelical angles with the program interhlx (K. Yap, University of Toronto) with residues 17–26 and 40–46 for the AB interhelical angle and residues 54–62 and 75–83 for the CD interhelical angle. The AB interhelical angle for cNTnC(L29Q) was 139 ± 5°, and the CD interhelical angle was 122 ± 7°, both of which are very close to the ORBplus prediction (143° and 118°, respectively) and to cNTnC (142 ± 3° and 109 ± 4°, respectively) (Table 1).

As trout cNTnC (ScNTnC) also contains Q29 in site 1, the structures of cNTnC(L29Q) and ScNTnC [61] were compared. The structural elements defined above were aligned between cNTnC(L29Q) and ScNTnC (Fig. 3A) and the RMSD was measured as 1.37 Å, not much different than that between cNTnC(L29Q) and cNTnC. This observation is supported by the interhelical angles (AB interhelical angle = 130 ± 3° and the CD interhelical angle = 112 ± 5°), indicating that Q29 does not impart a large-scale structural perturbation in the backbone of cNTnC.

To investigate whether Q29 has an effect on local structure, the conformation of sites 1 and 2 was examined more closely. Both sites were inspected since it has been shown that they are structurally and thermodynamically coupled [19]. The site 1 sequence alignment of cNTnC(L29Q), cNTnC, and ScNTnC is given in Fig. 2B. The structures were aligned at residues 15–27 (helix A) and 41–48 (helix B) and the orientation of the site 1 loop was contrasted between the structures (Fig. 3C). The Cα RMSD for the alignment of cNTnC(L29Q) with cNTnC was 3.20 Å and the Cα RMSD for the alignment of cNTnC(L29Q) with ScNTnC was 1.77 Å. On the other hand, when the structures were aligned at residues 54–64 (helix C) and 76–79 (helix D), the orientation of the site 2 loop was similar between all three isoforms (Cα RMSD for the alignment with cNTnC was 1.94 Å and the Cα RMSD for the alignment with ScNTnC was 1.78 Å). Therefore, although there is no global structural change induced by L29Q, there appears to be a minor perturbation limited to the direct vicinity of the mutation.

### 3.3 Dynamics and stability of cNTnC(L29Q) and cNTnC

**Fig. 4:**
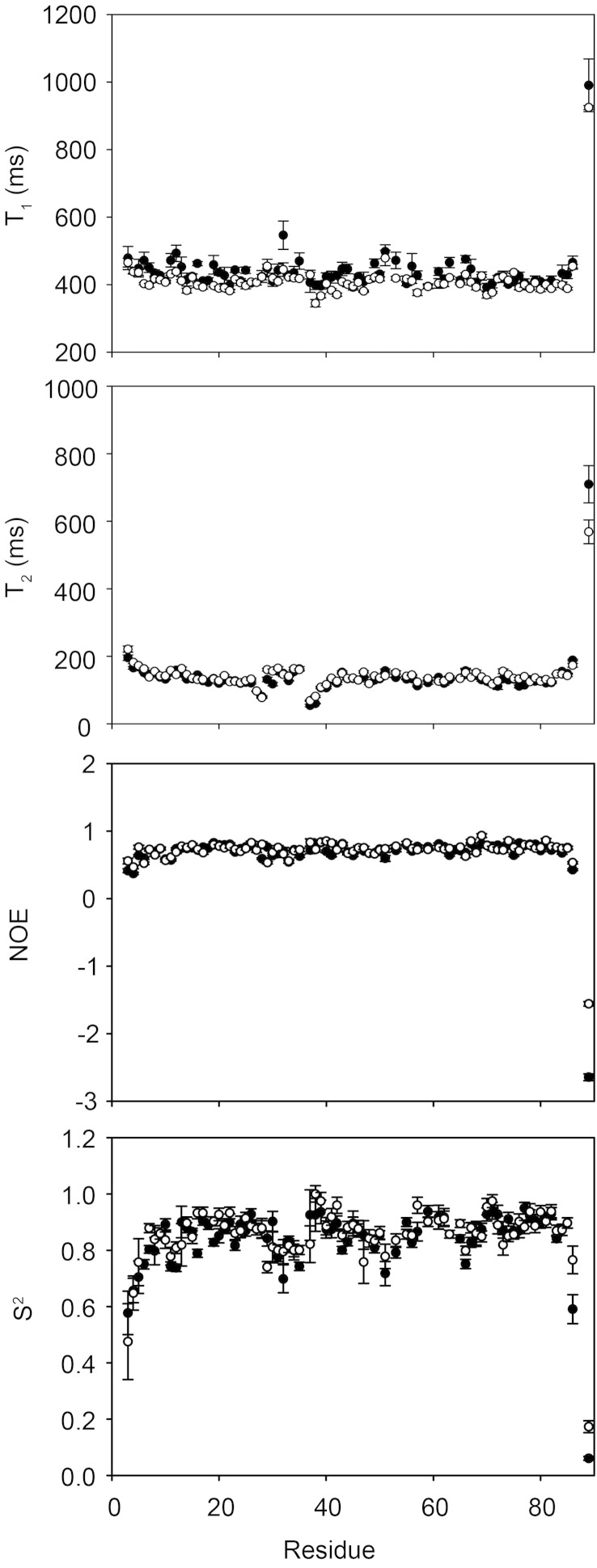
A comparison of T_1_, T_2_, NOE, and S^2^ for cNTnC(L29Q) (open symbols) and cNTnC (closed symbols). Relaxation data were collected at a magnetic field strength of 11.7 Tesla (^1^H larmor frequency of 500 MHz). Error bars denote SD.

To substantiate the small structural perturbation caused by L29Q, the backbone dynamics of cNTnC(L29Q) were compared with those of cNTnC. The ^15^N NMR relaxation parameters T_1_, T_2_, and NOE depend on the internal motions of the NH bond vector as well as on the overall tumbling, or rotational diffusion, of the protein and they can provide atomic level information on the dynamics of the protein. T_1_, T_2_, and NOE were measured for both cNTnC and cNTnC(L29Q) at 500 MHz (Fig. 4). Consistent with the lack of major structural changes, the NMR relaxation parameters did not vary significantly. The average relaxation parameters for cNTnC were: T_1_ = 440 ± 70 ms, T_2_ = 139 ± 69 ms, and NOE = 0.68 ± 0.39 and the average relaxation parameters for cNTnC(L29Q) were: T_1_ = 415 ± 63 ms, T_2_ = 144 ± 53 ms, and NOE = 0.70 ± 0.27. The values measured here were similar to those previously reported for cNTnC(wt), (T_1_ = 440 ± 106 ms, T_2_ = 166 ± 82 ms, and NOE = 0.57 ± 0.27) [19]. Correlation times (τ_m_) for cNTnC and cNTnC(L29Q) were calculated assuming the tumbling to be isotropic [19] based on T_1_ and T_2_ for residues with NOEs > 0.65, and were 5.64 ns and 5.26 ns for cNTnC and cNTnC(L29Q), respectively. These values are slightly higher than the theoretical τ_m_ (cNTnC: 5.09 ns; cNTnC(L29Q): 5.10 ns), which is most likely the result of concentration-dependent aggregation [53]. This small amount of aggregation is not expected to have a significant effect on the calculation of the order parameter (S^2^) [19]. Indeed, the average S^2^ of cNTnC was 0.834 ± 0.121 and the average S^2^ for cNTnC(L29Q) was 0.852 ± 0.108. Therefore, as was the case for the global conformation of cNTnC(L29Q) and cNTnC, both proteins also have similar overall backbone dynamics.

**Fig. 5:**
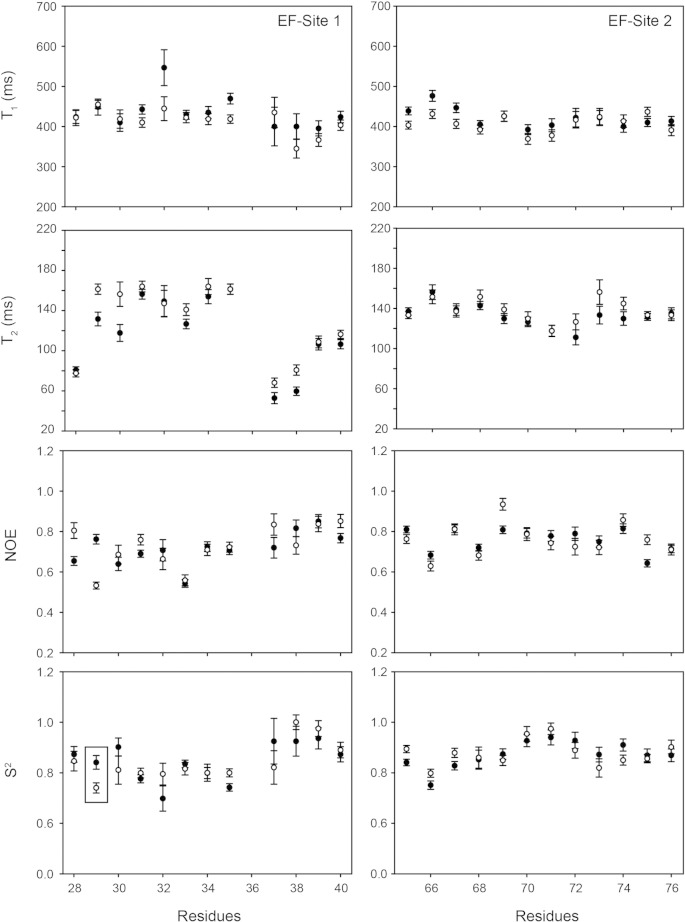
A comparison of T_1_, T_2_, NOE, and S^2^ for residues in sites 1 and 2 of cNTnC(L29Q) (open symbols) and cNTnC (closed symbols). Relaxation data were collected at a magnetic field strength of 11.7 Tesla (^1^H larmor frequency of 500 MHz). Residues 37, 38, 39, and 40 were fit with the S^2^-τ_m_-R_ex_ model. The S^2^ for residue 29 is highlighted by a box. Error bars denote SD.

Sites 1 and 2 were compared more closely to look for small localized perturbations, since these two sites are thermodynamically coupled [19]. The backbone dynamics of site 1 were very similar between cNTnC and cNTnC(L29Q) (Fig. 5); the average S^2^ was 0.844 ± 0.077 for cNTnC and the average S^2^ was 0.841 ± 0.077 for cNTnC(L29Q). However, there were local differences, primarily at residues near the mutation site. The order parameter of residue 29 indicated that Q29 is more flexible than L29 (S^2^ = 0.740 ± 0.020 versus 0.841 ± 0.027, respectively) and the combined ΔS^2^ of V28, Q29 and G30 were decreased by 0.22 ± 0.09 when compared with those of V28, L29, and G30. There was no major change in the dynamics of site 2, which is consistent with the structural alignment presented in the previous section. The average S^2^ for cNTnC was 0.872 ± 0.052 and for cNTnC(L29Q) it was 0.877 ± 0.051. Taken together, these data suggest that a localized structural effect of L29Q leads to an increase in the flexibility of part of site 1.

To determine whether this small change in structure and flexibility of site 1 translated to a change in stability, we used circular dichroism (CD) to follow the urea-induced denaturation of cNTnC or cNTnC(L29Q). Unfolding was monitored by measuring the CD signal at 222 nm (wavelength indicative of helicity) as a function of urea concentration (Supplementary Fig. 3). The [urea]_1/2_ values are very similar: 4.6 ± 0.1 M and 4.8 ± 0.1 M for cNTnC and cNTnC(L29Q), respectively, but the *m* values are slightly less for cNTnC(L29Q): 4.1 ± 0.1 *versus* 3.3 ± 0.4. This translates into a higher Gibbs free energy of unfolding (ΔG^0^_F-U_) for cNTnC (ΔG^0^_F-U_ = 18.9 ± 0.6 kJ mol^− 1^) than cNTnC(L29Q) (ΔG^0^_F-U_ = 15.8 ± 1.9 kJ mol^− 1^). Thus, in addition to altering the structure and dynamics of site 1, the L29Q mutation also slightly decreases the stability of cNTnC.

### 3.4 Prediction of the structure of cNTnC(L29Q) bound to cTnI

Although no large conformational changes were observed for cNTnC(L29Q) in comparison with cNTnC, it is possible that L29Q alters the conformation of cNTnC in the presence of its binding partners. For example, a recent computational study [31] proposed that while the structure of cNTnC-cTnI_147–163_ (as defined by the AB interhelical angle) was sensitive to the pseudo-phosphorylated state of cTnI_1–32_ (represented as S22D, S23D), that of cNTnC(L29Q)-cTnI_147–163_ was not. Moreover, that study suggested that cNTnC(L29Q) is more open than cNTnC; an AB interhelical angle difference of 5° when bound to cTnI_1–32_ and 13° when bound to cTnI_1–32(S23D,S24D)_ was calculated. To investigate this possibility, we used ORBplus to predict the conformation of cNTnC and cNTnC(L29Q) based on the NMR chemical shifts published in Baryshnikova et al. [32] (Table 1). The AB and CD interhelical angles were calculated for cNTnC(L29Q) and cNTnC in the following complexes: cNTnC, cNTnC-cTnI_1–29_, cNTnC-cTnI_1–29_PP (phosphorylated at S22, and S23), cNTnC-cTnI_147–163_-cTnI_1–29_, and cNTnC-cTnI_147–163_-cTnI_1–29_PP. In contrast with the computational study [31], we found no effect of cTnI_1–29_ (or cTnI_1–29_PP) on either the AB or CD interhelical angles. Furthermore, in the presence of cTnI_1–29_ (or cTnI_1–29_PP), we found that the AB angle of cNTnC(L29Q) is slightly more closed than that of cNTnC (109–111° versus 105–104°, respectively). Nevertheless, the angular changes are small, which is consistent with our interpretation that the small chemical shift perturbations induced by cTnI_1–29_ binding are likely to be the result of a local electrostatic interaction rather than a large conformational change of cNTnC [32].

### 3.5 Functional and structural impact of cTnC(L29Q) in cardiac muscle cells

The *in vitro* characterization of cNTnC(L29Q) by NMR spectroscopy indicated that the global conformation of cNTnC was not altered by the mutation, suggesting that this cardiomyopathy-causing mutation may exert its effect by altering the protein-protein interactions in the downstream signaling pathway. The structural and functional impact of L29Q was therefore investigated in demembranated ventricular trabeculae, in which the native assembly and interactions of the proteins are largely preserved. The *in situ* orientation of cTnC was determined using TnC mutants with BR attached along either the C helix (BR-cTnC_C_), adjacent to the regulatory Ca^2 +^-binding site, or the E helix in the C-lobe (BR-cTnC_E_) (Supplementary Fig. 4). Maximum Ca^2 +^-activated isometric force after introduction of cTnC(L29Q) was 31 ± 4 mN mm^− 2^ (SEM, n = 11) for BR-cTnC(L29Q)_C_ and 41 ± 5 mN mm^− 2^ (n = 10) BR-cTnC(L29Q)_E_. These values are not statistically different (P > 0.05) from those measured after replacement of native TnC by wild-type BR-cTnCs, 38 ± 4 mN mm^− 2^ (n = 12) for BR-cTnC_C_ and 36 ± 5 mN mm^− 2^ (n = 10) BR-cTnC_E_. Thus the introduction of L29Q did not significantly affect the maximum Ca^2 +^-activated force, in agreement with previous reports [27], [28], [29], [30]. Additionally, the slack length of the preparations after exchange of BR-TnC was the same as that without TnC exchange (sarcomere length 1.9 μm).

The orientation of the BR fluorescence dipole, and thus of the C or E helix of cTnC to which it was attached, was determined from the polarization of fluorescence from trabeculae containing BR-cTnC. The polarized fluorescence intensities were used to calculate the order parameter <* P*_2_ > that describes the orientation of the BR dipole [59]. <* P*_2_ > would be + 1 if all the probes were parallel to the trabecular axis, and − 0.5 if they were all perpendicular.

**Fig. 6:**
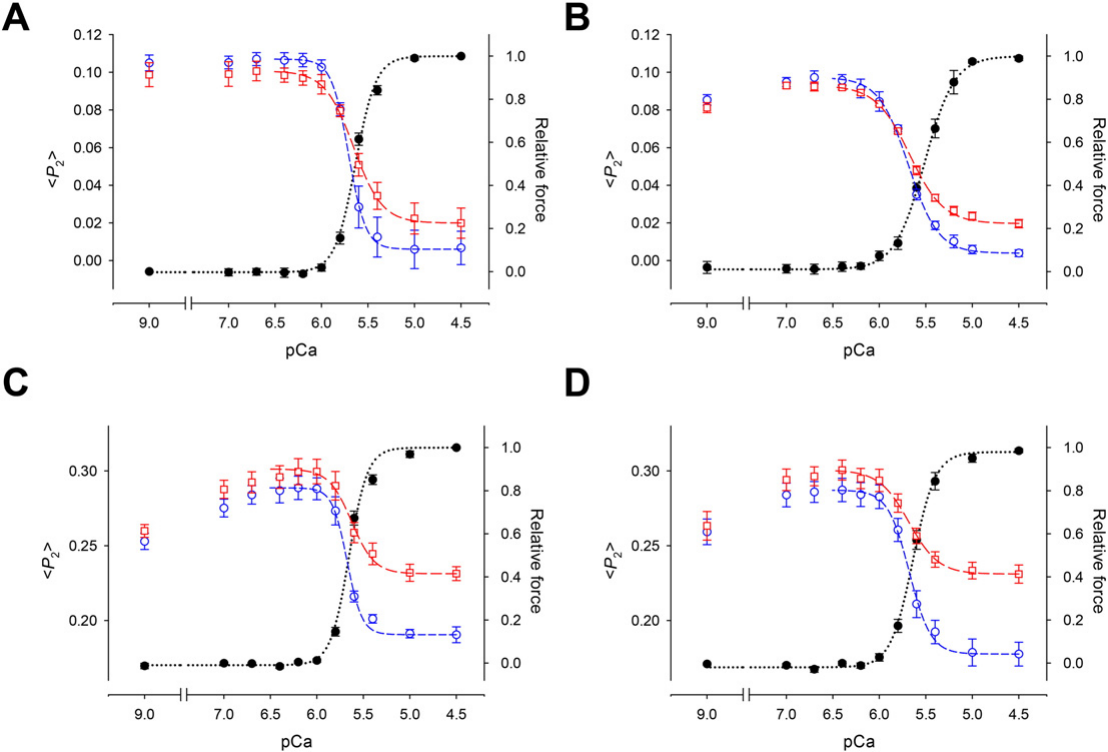
Ca^2 +^-dependence of < *P*_2_ > (open symbols) and force (filled circles) in trabeculae containing A. BR-cTnC_C_, B. cTnC(L29Q)_C_, C. BR-cTnC_E_ and D. BR-cTnC(L29Q)_E_. Normal trabeculae activation (blue circles) and activation with force inhibition by 25 μM blebbistatin (red squares). Dashed lines are fits of Hill equation to < *P*_2_ > and force, respectively. Error bars denotes SEM for n = 4–6 trabeculae.

The effect of the L29Q mutation on relationships between force or cTnC orientation (<* P*_2_ >) and free calcium concentration in the pCa (− log[Ca^2 +^]) range from 9.0 (relaxed) to 4.5 (full activation) was investigated using calcium titrations (Fig. 6). The data for both force and <* P*_2_ > for the C helix probe in the absence of L29Q (Fig. 6 A) were well fitted by the Hill equation, over the full range of pCa. However, in the presence of L29Q (Fig. 6B) <* P*_2_ > for the C helix probe showed an additional high-affinity component in the sub-threshold range of [Ca^2 +^], as observed for the E helix probe in both the absence ([37], Fig. 6 C) and presence of L29Q (Fig. 6D). The origin of the high affinity component is unknown, but since it is confined to the region in which no active force is generated, we confined all the Hill equation fits of <* P*_2_ > data to the region pCa 6.4 to pCa 4.5, in which they were well described by this equation (Fig. 6, dashed lines).

The effects of the L29Q mutation on the Hill parameters for both force and the orientation changes of the C and E helices of cTnC are summarized in Table 2. Here pCa_50_, the pCa for half-maximal change in either force or < *P*_2_ >, is a measure of Ca^2 +^ sensitivity; *n*_H_, the Hill coefficient, describes the steepness of the Ca^2 +^ dependence and is a measure of cooperativity of the Ca^2 +^-dependent change. The L29Q mutation was associated with a small but significant decrease in pCa_50_ for force in the trabeculae containing BR-TnC_C_, but there was no significant change for those containing BR-TnC_E_, and no significant effect on *n*_H_, although the mean value of the latter was lower in the presence of L29Q. Much clearer effects were seen in the fitted parameters for <* P*_2_ >, which is more reproducible between batches of trabeculae. The L29Q mutation had no significant effect on pCa_50_ for either the C or E helix probes, but in both cases it reduced *n*_H_, indicating a decrease in the cooperativity of the structural changes associated with calcium activation. The overall amplitude of the structural changes between pCa 6.4 and 4.5 was not affected by the presence of the L29Q mutation, consistent with the absence of a major change in the three-dimensional structure of the protein in the NMR results presented above.

### 3.6 cNTnC(L29Q) abolishes the myofilament response to force generating myosin heads

Strongly bound myosin cross-bridges that are formed in the absence of ATP (in rigor) can contribute to thin filament activation [62], [63], [64], and force-generating myosin cross-bridges in the presence of ATP can increase the Ca^2 +^ sensitivity of cTnC structural changes [37]. Furthermore, a recent report suggested that force-generating myosin heads are responsible for a Ca^2 +^-sensitizing effect on the opening of cNTnC [65]. These results indicate that force-generating myosin heads might stabilize the interaction between the exposed hydrophobic patch of cNTnC and the switch region of cTnI. We used 25 μM blebbistatin to abolish active force and thereby determine the effect of force-generating myosin heads on cTnC structure and calcium activation in the presence of the L29Q mutation. Inhibition of active force by blebbistatin reduced the amplitude of the changes in the orientation of both the C and E helices of cTnC as reported by <* P*_2_ >, in both the presence and absence of the L29Q mutation (Fig. 6 and Table 2). Blebbistatin also significantly (*P* < 0.05) reduced the steepness of the Ca^2 +^ dependence (*n*_H_, Table 2) independently of the presence of L29Q. In wild type cTnC, blebbistatin decreased pCa_50_ for both the C and E helix orientation (Table 2; P < 0.05, paired comparison), as reported previously [37]. Strikingly, L29Q abolished this effect of blebbistatin on pCa_50_ for the orientation changes of both helices; the effect of force-generating cross-bridges on the structural changes in cTnC associated with calcium activation is abolished by the L29Q mutation.

## 4 Discussion

FHC is the most commonly inherited form of cardiovascular disease [6], [7], and several FHC-related mutations have been identified in cTnC, including: L29Q [8], A8V, C84Y, E134D, D145E [66], Q122AfsX30 [67], and A31S [68]. Given that L29Q was the first FHC-linked mutation discovered in cTnC, it has garnered the most attention; however it remains unclear whether the mutation is pathogenic or simply a rare polymorphism. The only clinical instance of this mutation was in a 60-year-old patient [8], so it is likely that if L29Q is indeed pathogenic, the physiological effect is subtle. In this study, we attempted to characterize the *in vitro* and *in situ* structure and function of cTnC(L29Q) to understand its potential pathogenesis.

### 4.1 In vitro structure, dynamics and stability of cNTnC(L29Q)

NMR spectroscopy was used to solve the structure of the Ca^2 +^-bound conformation of cNTnC(L29Q). The overall structure was unperturbed by the mutation, resulting in an essentially closed conformation of cNTnC (Fig. 2 and Table 1). Closer inspection of the residues in the defunct site 1 revealed a slight perturbation the backbone atoms when compared to cNTnC (Fig. 3). Interestingly, the orientation of the site 1 loop is more similar to trout cNTnC (ScNTnC), which also contains a glutamine at residue 29. As a compensatory mechanism for its lower physiological temperature, the Ca^2 +^ affinity of trout cardiac troponin C (ScTnC) is higher than the mammalian isoform [69], [70]. Despite the dramatic difference in Ca^2 +^-affinity, the sequences of the N-domain of ScTnC (ScNTnC) and cNTnC have only five amino acid differences, four of which have been implicated as being primarily responsible for the increased Ca^2 +^ sensitivity: D2N, V28I, L29Q, and G30D [71]. One explanation for the change in structure may be that the mutation from a hydrophobic residue to a hydrophilic one destabilizes hydrophobic packing surrounding the residue. Indeed, this is supported by the increased flexibility of Q29 compared to L29 as indicated by the NMR relaxation measurement (Fig. 5). Urea-induced unfolding measurements indicated that the L29Q mutation caused a slight decrease in ΔG^0^_F-U_, which is consistent with a local structural destabilization of Q29. Recently, the Cd^2 +^-bound X-ray structures of several variants of cNTnC, including cNTnC(wt), cNTnC(L29Q), and cNTnC(NIQD) (D2N, V28I, L29Q, and G30D) have been published [34]. Although the numerous Cd^2 +^ molecules in the structures, including Cd^2 +^ bound to site 1, made the physiological relevance of these structures difficult to interpret, the authors also noted a small perturbation of 0.5 Å in the backbone of L29Q and 1.0 Å in the backbone of cNTnC(NIQD) in site 1 that is consistent with our findings. Together these data indicate that while L29Q does not drastically alter the structure of cNTnC, it does cause a small local rearrangement of site 1, which may alter its function, perhaps through hampering its interaction with other binding partners, such as cTnI (discussed below).

One limitation of the current study is that the structure does not include the C-domain of cTnC; therefore, we cannot rule out the possibility that L29Q elicits a structural perturbation in the C-domain and/or in the relative orientation between the N- and C-domains of cTnC *in vitro*. Indeed, an NMR study investigating the inter-domain conformation of cTnC indicated that while the two domains of cTnC are flexible, they preferentially adopt a compact structure [72] that is consistent with the X-ray structure of the cardiac troponin complex [73]. Although a long-range structural effect of L29Q *in vitro* is possible, our FISS data indicate that L29Q does not cause a significant perturbation on the orientation of the C-domain of cTnC *in situ* (Fig. 6 and Table 2).

### 4.2 Functional and structural impact of cTnC(L29Q) in cardiac muscle cells

To complement the studies at the isolated protein level described above, we used FISS to investigate the effects of L29Q on the structure of cTnC in its native sarcomere environment in rat ventricular trabeculae. The introduction of L29Q did not significantly affect maximum active force in this preparation, in agreement with previous reports [27], [28], [29], [30]. L29Q was associated with a significant decrease in the calcium sensitivity of active force as measured by pCa_50_ in trabeculae containing cTnC labeled with the C-helix probe, but the corresponding decrease for the E-helix probe was not significant at the 5% level. For both probes the mean value of the cooperativity parameter *n*_H_ for force was lower in the presence of L29Q but again the differences were not significant at the 5% level. Thus the present results cannot be regarded as conclusive on the effect of L29Q on the Ca^2 +^-dependence of active force, perhaps because this parameter depends on many factors downstream of cTnC and exhibits relatively high sample-to-sample variability. We therefore focused on the orientation changes of cTnC during Ca^2 +^ activation, which can be measured with greater reproducibility.

The structural changes of cTnC during Ca^2 +^ activation were determined from the polarization of fluorescence from trabeculae containing BR-labeled cTnC with the probe attached along either the C helix, adjacent to the regulatory Ca^2 +^-binding site, or the E helix, in the IT arm of the troponin complex. The polarized fluorescence intensities were used to calculate the order parameter <* P*_2_ > that describes the orientation of the BR dipole [59]. The Ca^2 +^-dependence of the <* P*_2_ > changes was described using the Hill equation, which gave a good fit to the C helix probe data over the full range of pCa (Fig. 6 A). However, in the presence of L29Q, <* P*_2_ > for the C helix probe showed an additional high-affinity component in the sub-threshold range of [Ca^2 +^] (Fig. 6 B), as observed for the E helix probe in both the absence ([37], Fig. 6 C) and presence of L29Q (Fig. 6 D). This additional component may be associated with Ca^2 +^ binding to the high-affinity Ca^2 +^/Mg^2 +^ sites in the C-terminal domain of cTnC. Since this component is observed at [Ca^2 +^] values below the physiological range, at which no active force is generated, we minimized its effect on the fitted Hill equation parameters by confining all the fits of the <* P*_2_ > data to the pCa region 6.4 to 4.5.

The overall amplitude of the structural changes in cTnC in the pCa region 6.4 to 4.5 was not affected by the L29Q mutation, which is consistent with the NMR results showing that L29Q does not markedly alter the structure of cNTnC. The L29Q mutation had no significant effect on pCa_50_ for either the C or E helix probe. This implies that, although cNTnC(L29Q) adopts a similar conformation as ScNTnC, it does not increase Ca^2 +^-sensitivity. This might be due to competing effects of the L29Q mutation on Ca^2 +^ sensitivity mediated by the interaction between cNTnC and the cardiac specific N-terminal region of cTnI (see below).

Force-generating myosin heads can increase the Ca^2 +^ sensitivity of structural changes in cTnC in the presence of ATP [37], possibly by promoting the opening of cNTnC [65]. The present study confirmed previous results [36] showing that inhibition of active force by blebbistatin reduced both the amplitude and the Ca^2 +^ sensitivity of the structural changes in cTnC (Fig. 6 and pCa_50_ in Table 2).

The present study showed that L29Q mutation reduced the steepness (*n*_H_) of the cTnC orientation changes induced by Ca^2 +^ (Table 2), indicating a decrease in the cooperativity of the Ca^2 +^-dependent structural changes. *n*_H_ was also reduced by adding blebbistatin, both in the presence and absence of the L29Q mutation, an effect that was not statistically significant in our previous study of the wild-type protein [37]. The difference is probably related to the use of a different calcium titration protocol in that study, in which each activation was initiated separately with relaxing solution, in contrast with the step-wise increments of [Ca^2 +^] used in the present study. The lower *n*_H_ observed in the previous work suggests that the cooperative activation of cardiac muscle cells may be influenced by the prior state of activation. In the present study any such effect was avoided by using the same protocol for the control and L29Q measurements.

In the presence of the L29Q mutation, inhibition of active force by blebbistatin reduced both the amplitude of the orientation changes in cTnC and the steepness of Ca^2 +^ dependence, as in wild-type cTnC. However the L29Q mutation abolished the effect of blebbistatin, and therefore the effect of force-generating cross-bridges on the Ca^2 +^ sensitivity of the structural changes (Table 2).

Taken together, the present results show that the L29Q mutation reduces both the cooperativity of Ca^2 +^-dependent structural changes in cTnC and the modulation of its Ca^2 +^ sensitivity by force-generating cross-bridges. These effects may be mediated by an altered interaction between cNTnC and the cardiac specific N-terminal region of cTnI, as discussed below.

### 4.3 cTnC(L29Q) alters the interaction between cNTnC and cTnI_1–32_

There have been numerous reports showing that the N-terminal region of cTnI interacts with cNTnC, both using fragments of cTnI [21], [22], [23], [24], [32], [74] and in the whole troponin complex [20]. When cTnI is phosphorylated at S22 and S23, its interaction with cNTnC is weakened [21], [22], [23], [24], [32] and Ca^2 +^-sensitivity is reduced (reviewed in [26]). An NMR study showed that the amide chemical shift of L29 is significantly perturbed when cTnI is phosphorylated, indicating that it plays a role in binding cTnI_1–32_ [23]. However, the interaction of the N-terminus of cTnI with cNTnC is weakened in the presence of the L29Q mutation, regardless of its phosphorylation state [24], [32]. This suggests that the pathogenesis of the L29Q may be manifested through an impaired interaction with the N-terminus of cTnI.

In the present study, amide chemical shifts were measured in a variety of cNTnC-cTnI and cNTnC(L29Q)-cTnI complexes (Table 1). Neither cTnI_1–29_ nor cTnI_1–29_PP caused major changes in the chemical shifts (and hence in the predicted conformation) of cNTnC or cNTnC(L29Q). These results are in contrast to those of a computational study of a model of the ternary complex of cNTnC-cTnI_147–163_-cTnI_1–32_ [31]. In that study, the AB inter-helical angle of cNTnC was increased by the presence of cTnI_1–32(S23D,S24D)_; but, in the same ternary complex containing cNTnC(L29Q), the presence of cTnI_1–32(S23D,S24D)_ had no structural effect. The increased AB inter-helical angle of cNTnC in the presence of cTnI_1–32(S23D,S24D)_ *versus* cTnI_1–32_ suggests that when cTnI_1–32_ is phosphorylated cNTnC is more closed and therefore is less likely to bind cTnI_147–163_ and promote contraction. Contrariwise, the lack of a change in the conformation of cNTnC(L29Q) between cTnI_1–32_ and cTnI_1–32(S23D,S24D)_ suggests that the L29Q mutation renders cTnC insensitive to this type of structural regulatory mechanism. The chemical shift data from our study, on the other hand, do not indicate a significant structural perturbation (as indicated by a change in the AB or CD inter-helical angles) induced by the N-terminus of cTnI, either upon binding cNTnC or cNTnC(L29Q) (Table 1). The peptide used in our study spans residues 1–29 and although this has been shown to the minimal length of cTnI required to elicit its modulatory role [75], it is possible that the extra residues not included in our work are necessary for the full structural effect of cTnI_1–32_. However, a recent study of cTnC bound to cTnI_1–73_, has indicated that the presence of the N-terminus of cTnI does not cause a structural perturbation of cNTnC [74]. So, it seems likely that cTnI_1–32_ alters contractility not through direct modulation of the conformation of cNTnC, but by another mechanism.

**Fig. 7:**
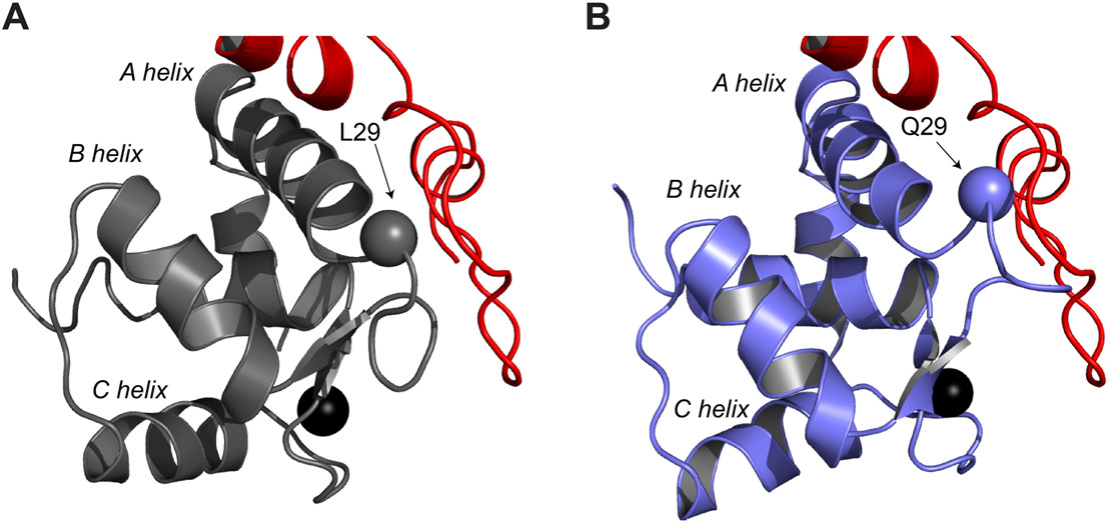
Model of the N-terminus of cTnI interacting with cNTnC and cNTnC(L29Q). The structures of A. cNTnC (gray) and B. cNTnC(L29Q) (slate) were aligned to cNTnC in a model of the thin filament that contained the N-terminus of cTnI (red) bound to cNTnC. The C_α_ of residue 29 is shown as a sphere. All structures are shown in cartoon representation and Ca^2 +^ ions are depicted as black spheres.

The NMR structure of cTnI_1–32_ [76] was recently used to construct a model of the interaction between cNTnC and cTnI_1–32_ [77], [78]. Although, it is likely that the N-terminus of cTnI does not bind to cNTnC in one single conformation [74], the model nevertheless provides utility in the interpretation of the wider structural implications of the L29Q mutation. In Fig. 7 we have superimposed the NMR structures of cNTnC (Fig. 7A) and cNTnC(L29Q) (Fig. 7B) with coordinates depicting the interaction between cNTnC and cTnI_1–32_. The model indicates that the site 1 loop from cNTnC(L29Q) clashes with backbone residues of cTnI_1–32_. This steric clash may explain the reduced affinity of the N-terminus of cTnI for cNTnC(L29Q) [24], [32]. Other explanations for the reduced affinity of cTnI_1–32_ for cNTnC(L29Q) may be the increased flexibility or decreased hydrophobicity of Q29, either of which could lead to a destabilization of the binding site(s) of cTnI_1–32_. Consequently, it is possible that the change in the conformation, dynamics and/or polarity of loop 1 contribute(s) to the pathogenesis of the L29Q mutation by impairing the cNTnC(L29Q)-cTnI_1–32_ interaction.

Our previous work showed that cNTnC moved towards the N-terminal region of cTnI during heart muscle activation, and that active force-generating myosin heads enhanced this structural transition [58]. In that model, the movement of cNTnC would enable its interaction with cTnI_1–32_ and pull the C-terminal region of cTnI (cCTnI) away from its actin-binding site. In addition, binding of myosin to actin during Ca^2 +^ activation would suppress the return of cCTnI to its actin-binding site by stabilizing tropomyosin away from its initial inhibitory position, consequently facilitating the movement of cNTnC and its interaction with cTnI_1–32_. The present results suggest that this interaction is linked to the cooperative activation of the heart muscle and its Ca^2 +^ sensitivity. Inhibition of active force by blebbistatin, on the other hand, would reduce the interaction between cNTnC and cTnI_1–32_ and, according to this hypothesis, the associated changes in cooperativity and Ca^2 +^ sensitivity.

The weakening of the interaction between cNTnC and cTnI_1–32_ by the L29Q mutation seen in previous studies might therefore act by weakening its stabilizing effect on the active state, associated with the decrease of cooperativity and Ca^2 +^ sensitivity. However, the decrease in Ca^2 +^ sensitivity produced by the TnI interaction seems to be compensated for by a local effect on Ca^2 +^ affinity, which would account for our finding that the L29Q mutation has no net effect on the Ca^2 +^ sensitivity of the structural changes in cTnC. On the other hand, the effect of the L29Q mutation on the structural changes in cTnC associated with force-generating myosin heads would have a profound impact on the cardiac function. Since the myofilament response to force-generating myosin heads is an important regulatory mechanism in cardiac muscle contraction, the lack of this coupling in the presence of L29Q mutation in cTnC could contribute to its pathogenesis in FHC.

## 5 Conclusion

In this study we use a variety of structural and functional techniques to shed light on the effect of the FHC-related L29Q mutation in cTnC. Our NMR spectroscopy data for the structure of the Ca^2 +^-bound cNTnC(L29Q) revealed that the overall structure was unperturbed by the mutation, although some small local changes were observed which translate to a slight decrease in protein stability. Our *in situ* polarized fluorescence results from heart muscle cells showed that, although the L29Q mutation did not affect the overall amplitude and Ca^2 +^-sensitivity of the cTnC structural changes, it decreased their cooperativity. Furthermore, the L29Q mutation abolished the effect of force-generating cross-bridges on the Ca^2 +^ sensitivity of the structural changes, which could contribute to the pathogenesis of FHC. This type of multidisciplinary structural study will be crucial in the future to construct a structural and functional paradigm for the etiology of inherited cardiomyopathies that target the sarcomere.

## Disclosures

None.

## Figures and Tables

**Table 1:** Interhelical angles of cNTnC.

	AB angle (°)	CD angle (°)	Ref.
*Experimental measurements*^a^			
cNTnC(WT)	134 ± 3	118 ± 4	[12]
cNTnC	142 ± 3	109 ± 4	[11]
cNTnC(WT)-cTnI_147–163_	102 ± 4	95 ± 6	[15]
ScNTnC	130 ± 3	112 ± 5	[61]
cNTnC(L29Q)^b^	139 ± 5	122 ± 7	–

*ORBplus calculations*^c^			
cNTnC(L29Q)^b^	143	118	–
cNTnC(L29Q)-cTnI_1–29_	143	117	[32]
cNTnC(L29Q)-cTnI_1–29_PP	143	118	[32]
cNTnC(L29Q)-cTnI_147–163_	106	85	[32]
cNTnC(L29Q)-cTnI_147–163_-cTnI_1–29_	109	87	[32]
cNTnC(L29Q)-cTnI_147–163_-cTnI_1–29_PP	111	93	[32]
cNTnC	144	117	[11]
cNTnC-cTnI_1–29_	144	116	[32]
cNTnC-cTnI_1–29_PP	145	117	[32]
cNTnC-cTnI_147–163_	106	93	[32]
cNTnC-cTnI_147–163_-cTnI_1–29_	105	91	[32]
cNTnC-cTnI_147–163_-cTnI_1–29_PP	104	91	[32]

a Residues 17–26 and 40–46 for the AB interhelical angle and 54–62 and 75–83 for the CD interhelical angle. Angles were calculated using interhlx (K. Yap, University of Toronto).

b This work.

c Calculated using chemical shifts from residues 27–40 for the AB interhelical angle and 64–74 for the CD interhelical angle. The amide chemical shifts of residue 29 were not used in the calculation for cNTnC(L29Q).

**Table 2:** Ca^2 +^-dependence of force and the cTnC orientation parameter <* P*_2_ >.

	BR-cTnC_C_	+ 25 μM blebbistatin	BR-cTnC_C_ (L29Q)	+ 25 μM blebbistatin	BR-cTnC_E_	+ 25 μM blebbistatin	BR-cTnC_E_ (L29Q)	+ 25 μM blebbistatin
(n = 4)		(n = 4)		(n = 5)		(n = 6)	
Force								
pCa_50_	5.64 ± 0.02		5.55 ± 0.03 ^#^		5.67 ± 0.02		5.63 ± 0.02 ^NS^	
*n*_H_	3.35 ± 0.18		2.88 ± 0.31 ^NS^		3.66 ± 0.29		3.34 ± 0.41 ^NS^	
<* P*_2_ >								
pCa_50_	5.72 ± 0.01	5.65 ± 0.01 *	5.69 ± 0.01 ^NS^	5.68 ± 0.01 ^ns^	5.69 ± 0.01	5.63 ± 0.01 *	5.67 ± 0.01 ^NS^	5.67 ± 0.02 ^ns^
*n*_H_	4.14 ± 0.25	2.66 ± 0.34 *	2.98 ± 0.14 ^#^	2.29 ± 0.10 *	4.20 ± 0.18	3.17 ± 0.27 *	3.37 ± 0.23 ^#^	2.46 ± 0.18 *
at pCa 6.4	0.106 ± 0.004	0.098 ± 0.004 *	0.098 ± 0.001 ^NS^	0.092 ± 0.001 *	0.287 ± 0.008	0.296 ± 0.008 *	0.287 ± 0.008 ^NS^	0.301 ± 0.007 *
at pCa 4.5	0.007 ± 0.009	0.020 ± 0.008 *	0.003 ± 0.003 ^NS^	0.020 ± 0.002 *	0.190 ± 0.005	0.232 ± 0.006 *	0.178 ± 0.008 ^NS^	0.231 ± 0.006 *

Mean ± SEM. pCa_50_ and *n*_H_ are fitted parameters of Hill equation (see Methods and Materials). Comparisons: with and without L29Q mutation (*t* test, two-tailed; ^NS^*P* > 0.05; ^#^*P* < 0.05); before and after addition of blebbistatin (paired *t* test, two-tailed; ^ns^*P* > 0.05; * *P* < 0.05).

## Footnotes

3 cNTnC(C35S,C84S) and the cTnC(C35S,C84S) will be referred to as cNTnC and cTnC, respectively.
