# Supplementary material for: The structural and functional effects of the familial hypertrophic cardiomyopathy-linked cardiac troponin C mutation, L29Q
Source: J Mol Cell Cardiol. 2015 Oct;87:257–69. doi: 10.1016/j.yjmcc.2015.08.017 (PMC4640586; doi:10.1016/j.yjmcc.2015.08.017)
Supplement: Supplementary file 1 — Supplementary material. [file mmc1.docx]

**Supplementary Material:**


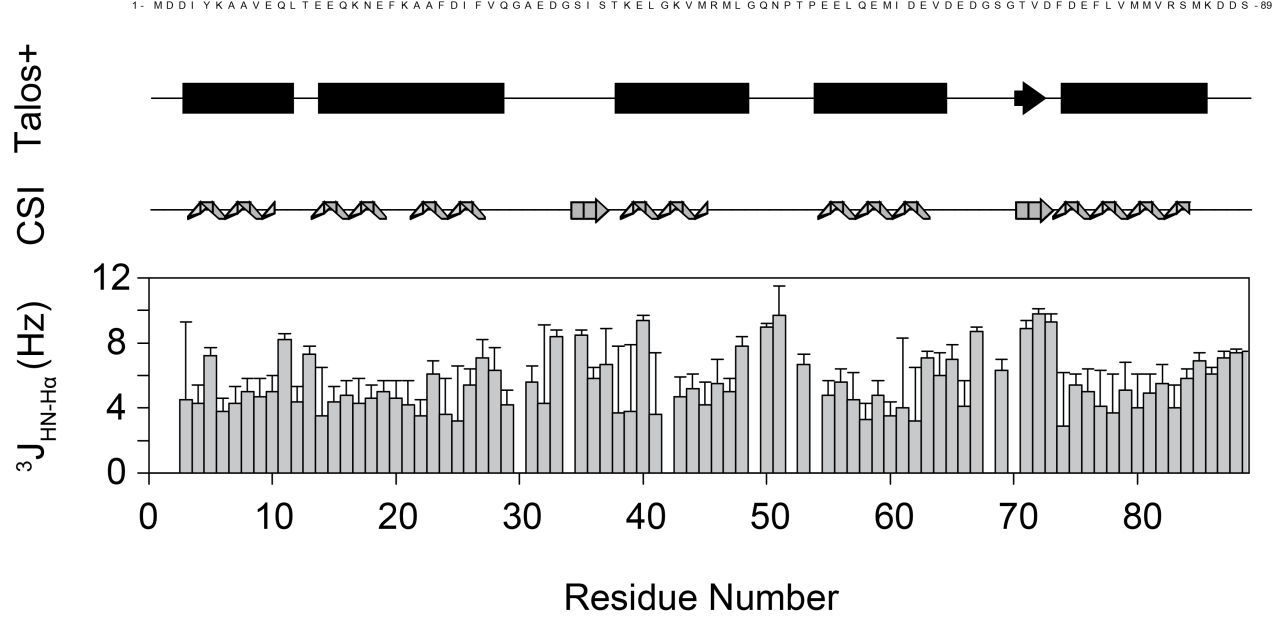


**Supplementary Figure 1.** Secondary structure features of cNTnC(L29Q) as predicted by Talos+ (α-helices are shown as rectangles and β-sheets as arrows) and the chemical shift index (using Hα, Cα, Cβ, and N; α-helices are shown as ribbons and β-sheets as arrows). Below is the ^3^J_HN-Hα_ values (coupling constant near 4 Hz are consistent with an α-helical structure, and values near 10 Hz are more consistent with a β-sheet). Overall, the different methods agree that cNTnC(L29Q) has 5 α-helices and 1-2 β-strands.


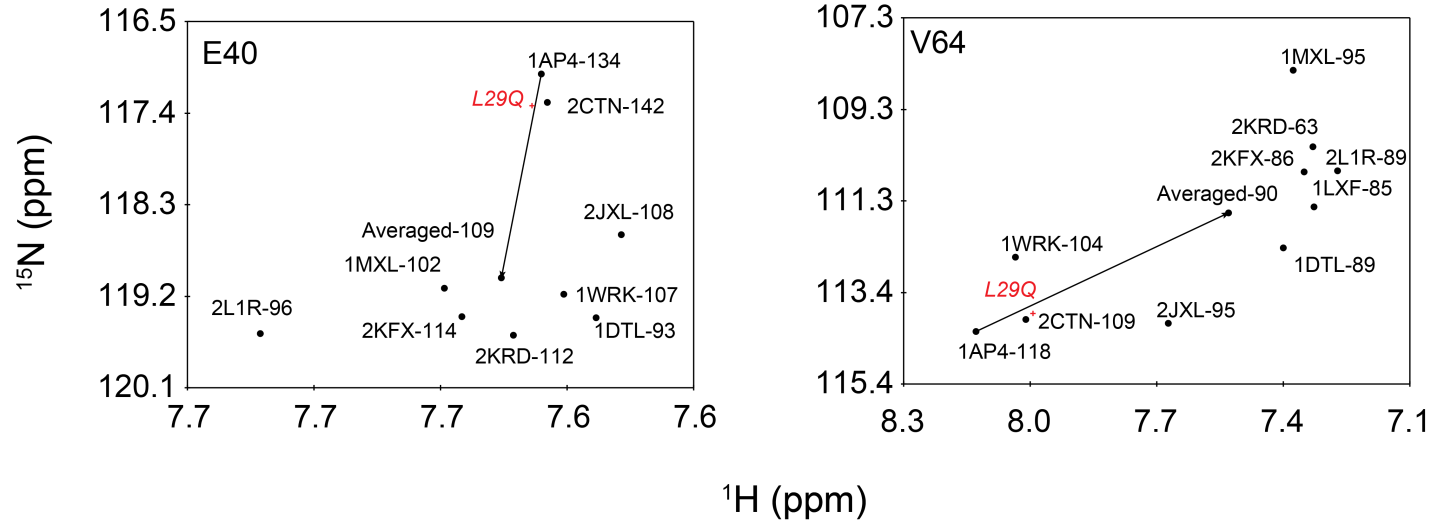


**Supplementary Figure 2.** ORBplus prediction of the interhelical angles of cNTnC(L29Q). The ‘hinge’ residues of cNTnC are illustrated above. The pdb codes and corresponding interhelical angles for the database proteins used in the calculation are shown, as well as the computed average chemical shift and average interhelical angle. The chemical shifts of L29Q are closest to 2CTN and 1AP4, which represent cNTnC(C35S,C84S) and cNTnC(WT), indicating that L29Q does not cause a significant structural perturbation in cNTnC.


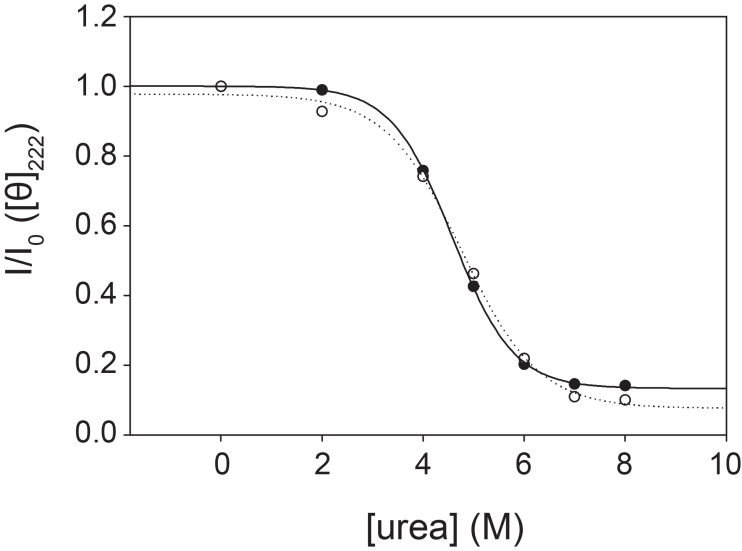


**Supplementary Figure 3.** Urea-induced unfolding of cNTnC (closed circles) or of cNTnC(L29Q) (open circles) monitored by CD at 222 nm at varying concentrations of urea. The data is plotted as the ratio of the CD signal at a given concentration of urea (I) over the CD signal for the native protein in the absence of urea (I_0_).


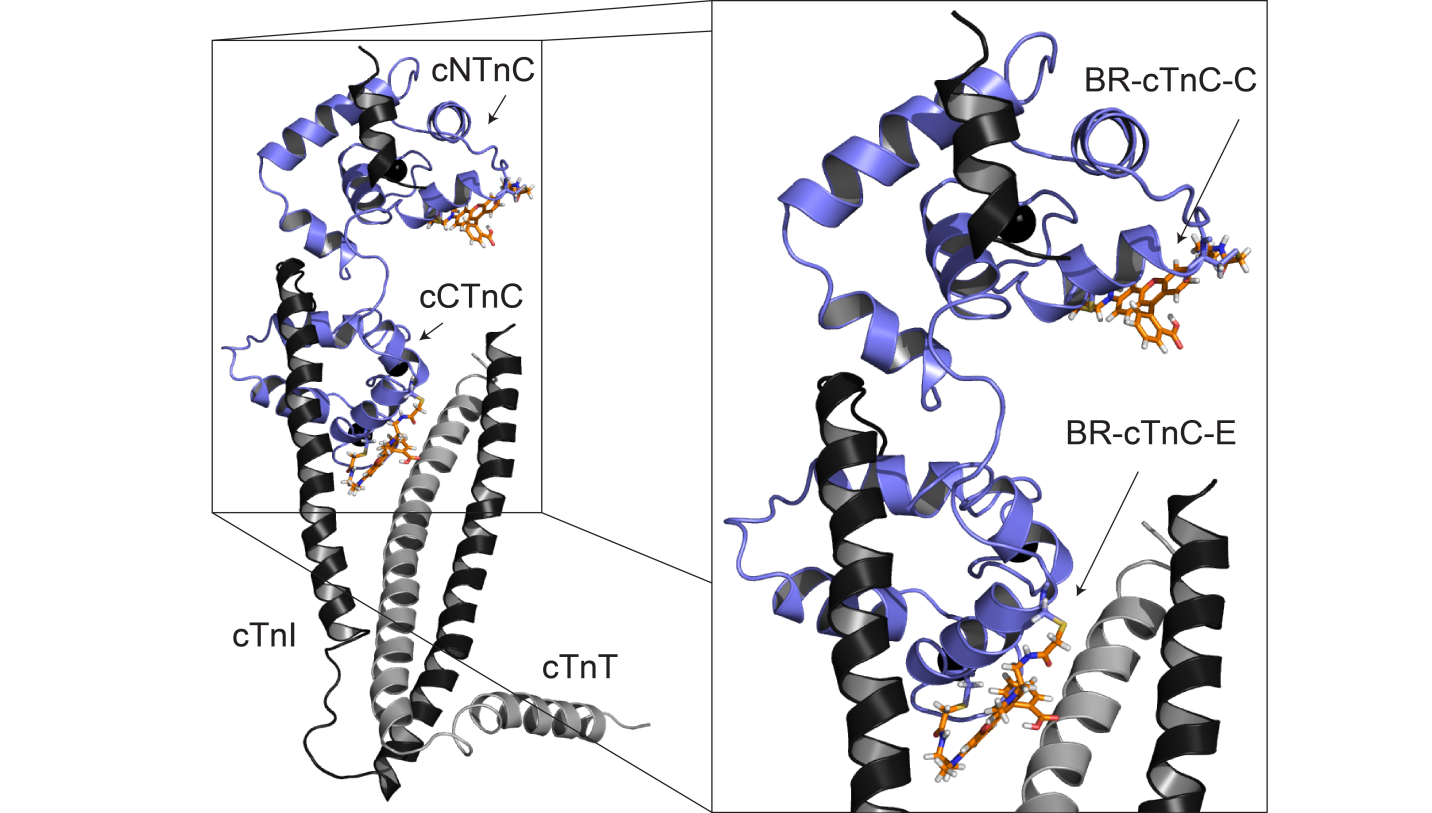


**Supplementary Figure 4.** Location of BR-probes on cTnC. All proteins of the troponin complex (PDB: 1J1D) are shown in cartoon representation: cTnC is colored in slate, cTnI is colored in black, and cTnT is colored in grey. Ca^2+^ is represented as black spheres and the BR probes are shown in stick representation.

**Supplementary Table 1.** NMR experiments and experimental parameters acquired for the structure calculation of cNTnC(L29Q):

| Experiment | nuclei | Field | nt | x-pts | y-pts | z-pts | x-sw^1^ | y-sw | z-sw | mix (s) |
| --- | --- | --- | --- | --- | --- | --- | --- | --- | --- | --- |
| Assignment |  |  |  |  |  |  |  |  |  |  |
| ^15^N-HSQC | ^1^HN,^15^N | 600 | 4 | 1024 | 256 | - | 8398 | 2432 | - | - |
| ^13^C-HSQC | ^1^H,^13^C | 600 | 8 | 1024 | 384 | - | 8398 | 12066 | - | - |
| HNCACB | ^1^HN,^13^C,^15^N | 600 | 16 | 1024 | 128 | 64 | 8398 | 12067 | 2431 | - |
| CBCA(CO)NNH | ^1^HN,^13^C,^15^N | 600 | 8 | 1024 | 128 | 64 | 8398 | 12067 | 2431 | - |
| HC(CO)NH | ^1^HN,^1^H,^15^N | 500 | 8 | 1024 | 256 | 64 | 8385 | 8398 | 2026 | - |
| C(CO)NH | ^1^HN,^13^C,^15^N | 500 | 16 | 1024 | 144 | 64 | 8385 | 10057 | 2026 | - |
| Distance Restraints |  |  |  |  |  |  |  |  |  |  |
| ^15^N-HSQC-NOESY | ^1^HN,^1^H,^15^N | 500 | 8 | 1024 | 256 | 64 | 8385 | 8398 | 2026 | 0.1 |
| ^13^C-HSQC-NOESY^2^ | ^1^H,^1^H,^13^C | 600 | 16 | 1024 | 128 | 128 | 8398 | 5999 | 4525 | 0.1 |
| ^13^C-HSQC-NOESY^3^ | ^1^H,^1^H,^13^C | 500 | 16 | 1024 | 144 | 48 | 8385 | 4999 | 3771 | 0.1 |
| Dihedral Restraints |  |  |  |  |  |  |  |  |  |  |
| HNHA | ^1^HN,^1^H,^15^N | 500 | 8 | 1024 | 180 | 64 | 8385 | 8398 | 2026 | - |
| HNHB | ^1^HN,^1^H,^15^N | 500 | 4 | 1024 | 256 | 64 | 8385 | 8398 | 2026 | - |
| HN(CO)HB | ^1^HN,^1^H,^15^N | 500 | 16 | 1024 | 128 | 64 | 8385 | 8384 | 2026 | - |

^1^sw all reported in Hz rounded to the nearest Hz.

^2^C-13 carrier set to 43 ppm.

^3^C-13 carrier set to 125 ppm.

**Supplementary Table 2:** *Structural statistics for 20 NMR structures of cNTnC(L29Q)*

|  | **Backbone atoms** | **Heavy Atoms** |
| --- | --- | --- |
| **R.m.s.d. from the average structure** |  |  |
| Ordered residues^a^ (Å) | 0.94 ± 0.18 | 1.40 ± 0.16 |
| **Total Distance Restraints** | 1692 | |
| **Intra Residual NOEs** | 1033 | |
| **Short range (*\|i-j\|*=1) NOEs** | 307 | |
| **Medium range (1<*\|i-j\|*<5) NOEs** | 191 | |
| **Long range (*\|i-j\|*≥5) NOEs** | 153 | |
| **Ca^2+^ distance restraints** | 8 | |
| **Dihedral restraints** | 175 | |
| **φ/ψ** | 154 (72/72) | |
| **χ_1_** | 21 | |
| **NOE violations/Structure^b^** |  | |
| **> 0.5 Å** | 0.0 | |
| **> 0.3 Å** | 0.0 | |
| **> 0.1 Å** | 3.35 | |
| **Dihedral Violations/Structure (> 5º)** | 0.0 | |
| **Ramachadran plot statistics^c^** |  | |
| **φ/ψ in most favored regions (%)** | 96.6 % | |
| **φ/ψ in additionally allowed regions (%)** | 3.4 % | |
| **φ/ψ generously allowed regions (%)** | 0.0 % | |
| **φ/ψ in disallowed regions (%)** | 0.0 % | |

^a^ Residues 3-49, 52-85; as calculated by psvs (http://psvs-1_4-dev.nesg.org/)

^b^ Violations are for the 20 NMR lowest energy structures

^c^ Procheck for ordered residues listed above [[79](file:///C:\Users\pmongcupa\AppData\Local\Temp\SALC7D.tmp\manuscript%20revision%20-2.docx#_ENREF_79)].

**References**

[79] R.A. Laskowski, J.A.C. Rullmann, M.W. MacArthur, R. Kaptein, J.M. Thornton, AQUA and PROCHECK-NMR: programs for checking the quality of protein structures solved by NMR, J. Biomol. NMR 8 (1996) 477–486.
